# Low Dimensional Carbon-Based Catalysts for Efficient Photocatalytic and Photo/Electrochemical Water Splitting Reactions

**DOI:** 10.3390/ma13010114

**Published:** 2019-12-25

**Authors:** Yoongu Lim, Dong-Kyu Lee, Seong Min Kim, Woosung Park, Sung Yong Cho, Uk Sim

**Affiliations:** 1 Department of Materials Science & Engineering, Chonnam National University, Gwangju 61186, Korea; youngun802@gmail.com (Y.L.); dk.lee2324@gmail.com (D.-K.L.); 2 Department of Bioengineering, School of Engineering, The University of Tokyo, 7-3-1 Hongo, Bunkyo-ku, Tokyo 113-8656, Japan; kimsmnice@gmail.com; 3 Division of Mechanical Systems Engineering, Institute of Advanced Materials and Systems, Sookmyung Women’s University, Seoul 04310, Korea; 4 Department of Environment and Energy Engineering, Chonnam National University, Gwangju 61186, Korea

**Keywords:** water splitting, electrochemistry, photoelectrochemistry, photocatalysis, carbon-based materials

## Abstract

A universal increase in energy consumption and the dependency on fossil fuels have resulted in increasing severity of global warming, thus necessitating the search of new and environment-friendly energy sources. Hydrogen is as one of the energy sources that can resolve the abovementioned problems. Water splitting promotes ecofriendly hydrogen production without the formation of any greenhouse gas. The most common process for hydrogen production is electrolysis, wherein water molecules are separated into hydrogen and oxygen through electrochemical reactions. Solar-energy-induced chemical reactions, including photocatalysis and photoelectrochemistry, have gained considerable attention because of the simplicity of their procedures and use of solar radiation as the energy source. To improve performance of water splitting reactions, the use of catalysts has been widely investigated. For example, the novel-metal catalysts possessing extremely high catalytic properties for various reactions have been considered. However, due to the rarity and high costs of the novel-metal materials, the catalysts were considered unsuitable for universal use. Although other transition-metal-based materials have also been investigated, carbon-based materials, which are obtained from one of the most common elements on Earth, have potential as low-cost, nontoxic, high-performance catalysts for both photo and electrochemical reactions. Because abundancy, simplicity of synthesis routes, and excellent performance are the important factors for catalysts, easy optimization and many variations are possible in carbon-materials, making them more attractive. In particular, low-dimensional carbon materials, such as graphene and graphitic carbon nitride, exhibit excellent performance because of their unique electrical, mechanical, and catalytic properties. In this mini-review, we will discuss the performance of low-dimensional carbon-based materials for water splitting reactions.

## 1. Introduction

Increasing energy consumption rates and dependency on fossil fuels have resulted in wide-spread ecological contamination. The dependency on fossil fuels can be reduced by encouraging the use of hydrogen as an energy resource. Hydrogen has the potential to be an ideal energy carrier because it theoretically possesses high gravimetric energy density, can be produced easily through various methods, and, most significantly, produces zero CO_2_ emission rates. Hence, the use of hydrogen as an energy source has the potential to significantly lower current global pollution rates. In addition, water splitting systems that involve splitting of water into hydrogen and oxygen molecules can be considered as sustainable and reliable because they can produce high-purity hydrogen and oxygen without any destructive by-products. Moreover, these sustainable water-splitting systems can be powered by existing renewable energy sources such as solar, wind, hydro, and geothermal powers. Since electrochemical water splitting was first reported, it has been widely applied. Water electrolysis, an efficient and clean technology used for the generation of high-purity hydrogen, also shows excellent adaptability. Solar-powered water splitting systems are developed through the engagement of photocatalytic and photoelectrochemical systems that effectively convert incident solar energy into chemical bonds by using light-absorbing materials [1,2,3,4].

Hydrogen is regarded as a clean solar fuel because of its high free energy content, accessibility as a water resource, and production of water and oxygen as sole byproducts [5]. Additionally, hydrogen can be stored and transported easily. Currently, hydrogen is produced on a large scale by reforming hydrocarbons or through the gasification of fossil fuels, both of which produce a large amount of carbon dioxide (CO_2_) [6]. The production of hydrogen from water splitting is a feasible approach to obtain an environment-friendly energy source. Water splitting is composed of two half-reactions, namely, hydrogen evolution reaction (HER) and oxygen evolution reaction (OER) [1,4,7], described by the following equations:

(1) $$\mathrm{OER}:\;2H_{2}O+4h^{+}\;↔\;4H^{+}+O_{2}E^{0}{}_{ox}=\;-1.23\;V\;\mathrm{vs}.\;\mathrm{RHE}$$

(2) $$\mathrm{HER}:\;4H^{+}+4e^{-}\;↔\;2H_{2}\;\;E^{0}{}_{red}\;=\;0.00\;V\;\mathrm{vs}.\;\mathrm{RHE}$$

**Figure 1:**
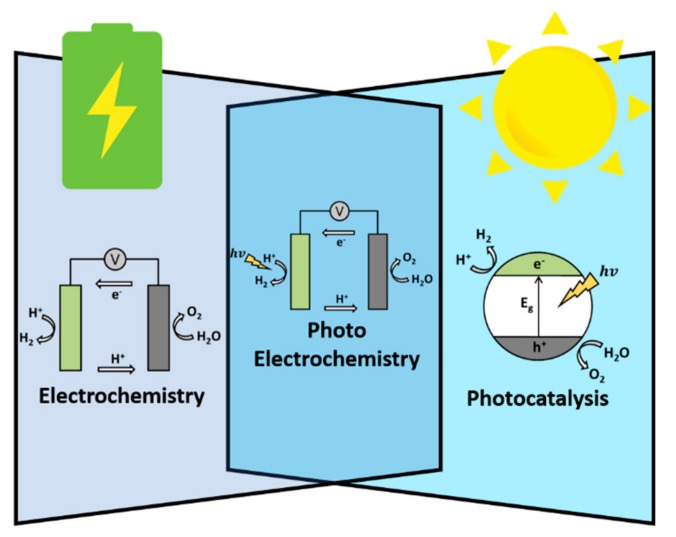
Schematic illustration of the three water splitting methods.

The methods of water splitting systems can be separated to electrochemistry, photoelectrochemisty, and photocatalysis, like shown in Figure 1. For the electrolysis of water which is shown in the left portion of Figure 1, the standard oxidization potential of the OER is defined as 1.23 V corresponding to a relative hydrogen electrode (RHE) and the reduction potential of HER is 0 V (vs. RHE) [6]. However, in the practical water splitting process, a larger applied potential is required because of disadvantageous factors such as activation energy, ion, and gas diffusion as well as factors related to the device. This additional potential is called overpotential (η) [5]. Many studies have been conducted with the aim of finding suitable electrocatalysts that could significantly decrease this overpotential and promote the reaction rate so as to enhance the total cell efficiency [2,3].

Numerous semiconductor materials have exhibited the capability to split water under solar irradiation [8,9]. The simplest photocatalytic water splitting process, shown in right portion of Figure 1, is accomplished through the use of a single semiconductor loaded with co-catalysts as surface sites for redox reactions [10,11]. By absorbing the photon of solar irradiation, the electrons in the valence band (VB) of the semiconductor are excited to the conduction band (CB), generating electron-hole pairs (EHPs) [12]. The generated EHPs migrate to the surface sites before any catalytic reactions can occur if the potentials of the CB and VB fulfill the requirements for water reduction and water oxidation. According to the values of water oxidation and reduction potentials, a minimum bandgap energy of 1.23 eV or larger is required to split water [13]. However, in practice, a slightly larger bandgap is required due to overpotential-related losses. Photocatalytic water splitting systems are extremely simple and have lower operating costs; however, the limited efficiency of the conversion of sunlight into chemical energy due to poor charge separation and charge recombination, along with a lack of light absorption, remains a challenge to be resolved [5,14].

The PEC cell, the center portion of Figure 1, is another promising device for use in solar-driven water splitting reactions [5]. The main component of the PEC cell is the semiconductor photoelectrodes, which convert incident photons to EHPs. These electrons and holes are spatially separated from each other due to the presence of an electric field inside the semiconductor. The photogenerated electrons are swept toward the conducting back contact and are transported to the counter-electrode via an external wire [13]. Similarly, to EC, the photocathode reduces water into hydrogen, whereas photoanode oxidizes water into oxygen. The challenge remains to design a PEC device with both a highly active photoanode and photocathode [15]. Half-reaction PEC cells, composed of noble-metal-based counter electrode along with a photocathode/photoanode, are more practical as these counter electrodes are usually highly active for water splitting reactions [6,14,16].

Carbon-based materials have attracted considerable attention for use in a variety of chemical reactions, such as HER, OER, ORR owing to their low cost, high abundancy and highly stable catalytic properties [8,9,17,18]. Additionally, the low-dimensional structure of carbon-based materials, including graphene [19,20], graphene oxide (GO) [12,21], reduced graphene oxide (rGO) [22], graphitic carbon nitride (g-C_3_N_4_) [23,24], graphene quantum dots (GQDs) [25,26], and graphene quantum sheets (GQSs) [27,28], has prompted intensive interest in photocatalysis, PEC, and EC [29]. Two-dimensional (2D) layered materials, such as graphene and its family, can act as “the building blocks” for the catalysts with their ultrathin plane structure [20]. These materials are “built” and held together by van der Waals force, and, by controlling the interlayer distance, the catalytic reactions are significantly accelerated [11,30]. GQDs and GQSs are zero-dimensional carbon materials that have been attracting huge interest due to merits, including analogous structure framework and constituent units to graphene, increment of active sites, and quantum confinement effects [25]. The bandgap and the alignment of band edges of these low-dimensional materials can be easily controlled by their thickness, atomic arrangement, functionalization, or in combination with other materials. In this review, we summarize the low-dimensional carbon-based materials for the EC, PEC, and photocatalytic water splitting system [7,31].

## 2. Low Dimension Carbon-Based Materials

### 2.1. Graphene

Graphene is a monoatomic layer of an indefinite number of carbon atoms arranged in a 2D hexagonal lattice [9]. Graphene provides a non-bandgap semiconductor material with high mobility and conductivity. The hexagonal ring of sp2 hybridized carbon atoms is comprised of three strong in-plane sigma bonds and one pi bond perpendicular to the planes. The perpendicular pi(π) bond enables graphene to form graphite, which is composed of multiple layers of graphene through van der Waals forces between layers [29]. With its unique mechanical, optic, electric, and thermal properties [32], graphene has shown favorability in PEC reactions. With regard to thickness, graphene is approximately 100 times stronger than the strongest steel [33]. Nearly transparent graphene has a white-light absorption rate that is approximately 2.3%, with an ability to transmit 97.7% of incident light [32]. This optical property is well suited for use as a co-catalyst in photocatalysis and PEC systems as the incident light directed towards the photocatalyst and photoelectrode is largely unimpaired. Due to its 2-dimensional structure, graphene is the only form of carbon in which every atom is available for chemical reaction on both sides [34]. Its large exposed surface area of 2630 m^2^·g^−1^ [33,35] coupled with its 2D structure acts as an excellent platform for photoelectrochemical applications. High electronic mobility, up to 200,000 cm^2^·V^−1^·s^−1^ [33], enables charge transfers during redox reactions, especially for HER. Graphene is an excellent thermal conductor which is also well known for its remarkable mechanical stability and electrical conductivity [20,35,36,37].

**Figure 2:**
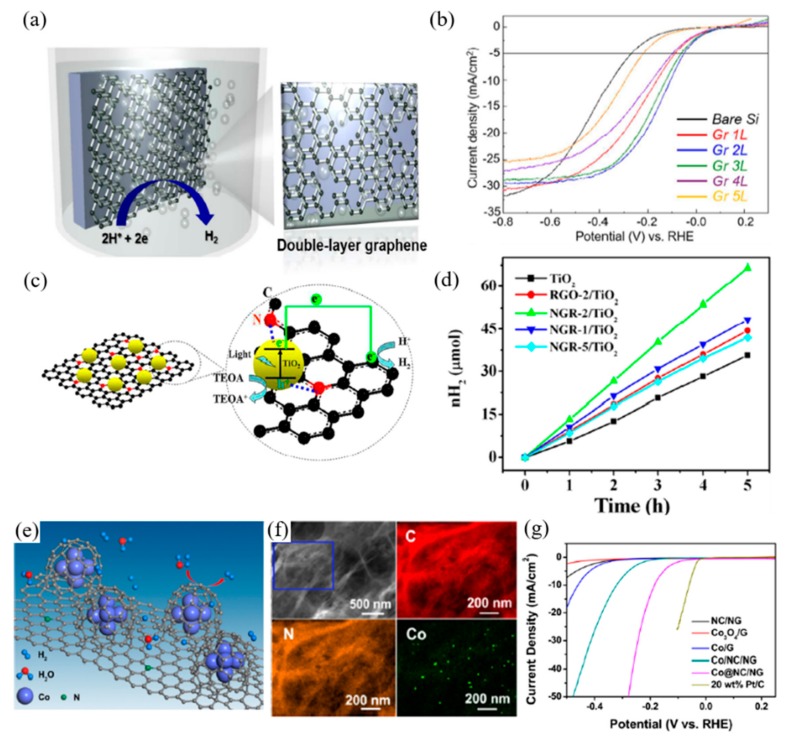
Graphene applied in photoelectrochemical cells (**a**,**b**), as photocatalysts (**c**,**d**), and electrochemical cells. (**e**–**g**) for water splitting reactions. (**a**) Schematic of double-layer graphene on p-silicon photocathode, (**b**) Photocurrent density–potential (J–E) curves of multiple layers of graphene on lightly boron-doped planar p-Si [39], (**c**) Schematic illustration for the strong coupling between TiO_2_ and N atoms in N-doped graphene sheets (NGR), enhanced photoinduced charge transfer, and photocatalytic hydrogen generation, (**d**) The time course of hydrogen production from a 50 mL aqueous solution containing 10 vol% TEOA aqueous solution with different photocatalysts [42], (**e**) Schematic representation of the HER process at the surface Co/NGR, (**f**) EDS elemental maps of C, N, and Co for Co/NGR, (**g**) Polarization curves for HER in 0.5M H_2_SO_4_ at a glassy carbon electrode modified with indicated catalysts [44].

Graphene has been applied to photocatalytic water splitting reactions as well as PEC systems due to its excellent conductive properties and large surface area. As a co-catalyst, N-doped monolayered graphene has been employed on Si photocathodes so as to investigate the catalytic activity of graphene and the effect of nitrogen doping in the PEC system. Sim et al. [38] have transferred monolayers of N-doped graphene to the surface of Si photocathodes following their growth on Cu foil. Compared to both the untreated Si photocathode and the photocathode with the undoped graphene layer, nitrogen doping showed an improvement in PEC performance with a significant positive shift in the onset potential without any changes in the saturated current density. Subsequently, the group investigated the layer dependence of graphene in HER catalysis. As shown in Figure 2a,b, a double-layer of graphene exhibits a greater anodic shift of the onset potential, thereby increasing the photon-to-current conversion efficiency. The analysis shows lower charge-transfer resistance and increments of the junction effect in the double-layer graphene/Si structure, compared to the structure with mono-/multi-layer graphene [39].

Owing to its outstanding electrical properties, graphene can effectively improve the charge separation and suppress the recombination of the excited carriers, generated by the photocatalysts or photoelectrodes. Lv et al. [40] investigated the synergetic effect of graphene with TiO_2_ and Cu for improved photocatalytic reaction. While the TiO_2_ generates electron-hole pairs under light irradiation, charge carriers are efficiently separated due to the high charge carrier transfer and mobility of graphene. PEC measurement also demonstrates an increment of photocurrent density with graphene as a co-catalyst, compared to ones without graphene. When coupled with graphene, enhancement of PEC performance of the photoelectrode materials with low diffusion lengths and low absorption rates, such as Fe_2_O_3_, was witnessed. The high conductive properties of graphene compensate for the loss of efficiency caused by low diffusion lengths. Yoon et al. [41] reported on graphene grown in an inverse opal structure and loaded with Fe_2_O_3_. The composition of Fe_2_O_3_ and inverse opal graphene reached a photocurrent density of 1.62 mA·cm^−2^ at 1.5 V vs. RHE, under AM 1.5 G, which is 1.38-fold higher than that of pristine Fe_2_O_3_ with 1.17 mA·cm^−2^. The inverse opal structure of the graphene can also play an important role in the charge transferring mechanism where it facilitates the fast transfer of electrons, generated by Fe_2_O_3_ loading.

Chemical doping of heteroatoms such as boron and nitrogen is one of the most practical approaches to modify the diverse properties of graphene. Doping can cause semiconducting behaviors in graphene with different valence electrons of the heteroatoms. Additionally, the catalytic activity of graphene towards photocatalytic reactions is enhanced due to the formation of active regions caused by asymmetric spin and charge distributions during doping. Doping with nitrogen has been considered as a means of producing an ideal photocatalyst. Nitrogen-doped graphene (NGR) has shown great potential in metal-free photocatalysts. Along with their unique semiconducting and catalytic properties, it can also be used as a supporting matrix of semiconductor-based photocatalysts in enhancing photocatalytic activity. Mou et al. [42] have synthesized NGR functionalized with TiO_2_ nanoparticles through a solvothermal treatment approach. Photocatalytic reaction and PEC performance were measured to investigate the role of the NGR. NGR/TiO_2_ composite showed a photocurrent density of 7.0 μA·cm^−2^, whereas the TiO_2_ was about 1.5 μA·cm^−2^. The photocurrent density of TiO_2_ composite with reduced graphene oxide (rGO), which was also investigated as a comparison, was approximately 4.5 μA·cm^−2^, which demonstrates that NGR has a higher charge transfer rate than rGO. This high charge transfer rate facilitates the efficient generated-charge separation and disables charge recombination for enhanced hydrogen evolution.

The electrochemical study of modified NGR with a transition metal was conducted for the overall water splitting reactions. Joy et al. [43] incorporated nickel on NGR nanoribbons, schematics shown in Figure 2e, to enhance the electrochemical OER. Through one-pot synthesis, nickel nanoparticles are randomly distributed on to the NGR. The nitrogen and nickel dopants open the active centers on graphene, thus improving electrocatalytic reactions. The Ni-NGR composite improved the catalytic reaction with a Tafel value of 62 mV·dec^−1^ and a highest current density of 120 mA·cm^−2^ at 1.75 V vs. RHE. Zhou et al. [44] wrapped cobalt nanoparticles with nitrogen-doped carbon on NGR for efficient hydrogen production. In the first step of synthesis, cobalt precursor and cyanamide were adsorbed onto a layer of graphene oxide, and, through thermal decomposition of cyanamide and reduction of Co, N-doped carbon is wrapped over Co nanoparticles. An onset potential of −49 mV vs. RHE was achieved by the Co/NGR with an overpotential of 10 mA cm^−2^ which is approximately ~200 mV with a Tafel value of 79.3 mV·dec^−1^.

### 2.2. Graphene Oxide (GO)/Reduced Graphene Oxide (rGO)

Zero bandgaps of graphene have proved challenging for their use as a photocatalyst. Efforts have been made to overcome this challenge by modifying and manipulating graphene so as to activate its surface for reactions. Graphene oxide is a derivative of graphene, tuned according to the degree of oxidation. With an extended wavelength of light absorption, graphene oxide can be used in photocatalysis for H_2_ production, CO_2_ reduction, and pollutant degradation [12,29]. Due to graphene’s zero band-gap, electron-hole pairs generated through illumination, instantaneously degenerate through recombination. Therefore, a band-gap opening is necessary in converting metallic graphene into a defective semiconductor graphene [45]. Oxidized graphene, containing oxygen functionalities, behaves as a p-type semiconductor due to lower electron mobility [35]. Oxygenated functional groups transform conductive sp2 carbons of graphene into nonconductive sp3 bonds Resulting in the creation of the band-gap opening an increase in the oxygen content stimulates the formation of sp3 carbon among the graphitic sp2 carbons, thereby enlarging the bandgap opening. The bandgap of graphene oxide has been determined to be between 2.4~4.3 eV [9]. With the increment of oxygen contents, the valance band’s maximum position gradually changes while the position of the conduction band minimum remains unchanged. Due to their tunable properties, graphene oxide sheets exhibit multiple functionalities along with remarkable performance in solar-driven water splitting reactions [21].

**Figure 3:**
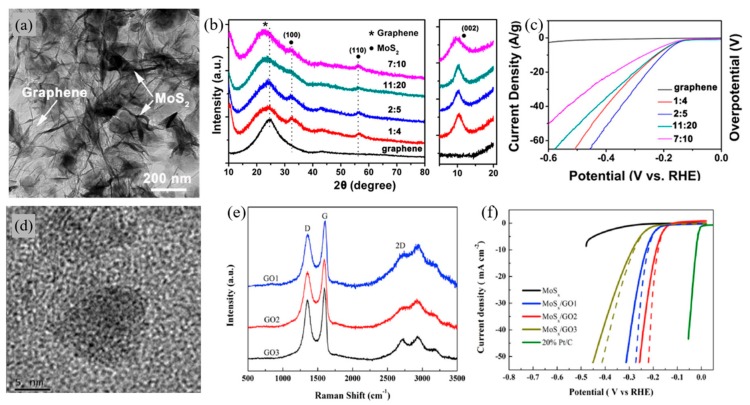
Graphene oxide (GO) applied in electrochemical cells for water splitting reactions. (**a**) Representative TEM image of the MoS_2_/Graphene, (**b**) XRD patterns of the MoS_2_/graphene hierarchical frameworks at different MoS_2_ loadings and zoom-in of the low-angle region of the XRD profiles, (**c**) Polarization curves of MoS_2_/graphene 3D framework at different MoS_2_ loadings. The currents were normalized to the total mass of the catalysts [46]. (**d**) HR-TEM image of the MoS_x_/GO nanocomposite, (**e**) Raman spectra of different GO, (**f**) Linear sweep voltammogram (LSV) curves in 0.5M H_2_SO_4_ for different MoS_x_/GO electrocatalysts [47].

With its large specific surface area and excellent electrical properties, graphene oxide is regarded as being an ideal support for catalysts. Zhou et al. [46] have grown MoS_2_ on graphene oxide as a hierarchical framework electrocatalyst for HER. The MoS_2_/GO framework hydrogels were prepared with the use of a simple hydrothermal process for a binder-free synthesis. This 3D hierarchical framework of MoS_2_/GO showed exhibited a specific surface area and electrical conductivity due to the π–π interaction between 2D layer properties of both MoS_2_ and GO. With a ratio of 2:5, MoS_2_ and GO respectively, showed superior catalytic activity towards HER with an overpotential of 107 mV, which is comparatively higher than that of pure GO, as shown in Figure 3b. The Tafel slope of approximately 86.3 mV·dec^−1^ was achieved with a 2:5 ratio. Moreover, the reaction exhibited a high level of stability with little or no change in the cathodic current during 8 h of continuous operation. The overall EC performance of the composite was superior to previous MoS_2_ catalyst’s performance, suggesting that GO is a well-suited framework for electrocatalysts. Hu et al. [47] have also supported MoS_x_ on GO with different degrees of oxidation through the use of facile wet chemical methods as an electrocatalyst for HER. Appling different weight ratios of graphite and KMnO_4_, GO, with various degrees of oxidation, was measured with Raman spectroscopy, as shown in Figure 3c. Peaks at ~1350, 1590, and 2700 cm^−1^ indicate the D, G, and 2D bands of graphene, respectively. The relative intensity ratio of I_D_/I_G_ shows the degree of oxidation. MoS_x_/GO composite with 1:4 ratio of graphite and KMnO_4_, respectively, denoted as MoS_x_/GO2 with I_D_/I_G_ ratio of 0.88, showed improved HER performance, as shown in Figure 3d. A composite with GO_2_ exhibited the best performance improvement with an overpotential of 180 mV and a Tafel value of 60.5 mV·dec^−1^. The MoS_x_/GO2 composite has the smallest charge transfer resistance value among all of the prepared samples, owing to the rapid electron transport between the active site and the electrode. It should be noted that by modifying the degree of oxidation in GO, catalytic performance can be enhanced.

Reduced-Graphene Oxide (rGO) occurs when graphene oxide is reduced for the purposes of increasing the quantity of active sites available for photoelectrochemical reactions [48]. rGO is known to suppress charge recombination and enhance charge separation owing to its excellent electrical properties [22]. Additionally, oxygenated functional groups, like GO, have shown an increment of active sites for chemical reactions in water splitting. With a bandgap range of 1~1.69 eV, depending on the degree of reduction, rGO can form a heterojunction with other light-absorbing materials, thereby enhancing charge separation. With excellent conductive properties, rGO plays a crucial role in the PEC system, as well as EC systems. As mentioned above, the photoanode collects the hole for OER while the electrons are collected by the photocathode for the HER in the EC/PEC system. The improved charge separation with rGO suppresses the surface recombination of photogenerated charge carriers, thereby, enhancing the overall water splitting reactions [29].

**Figure 4:**
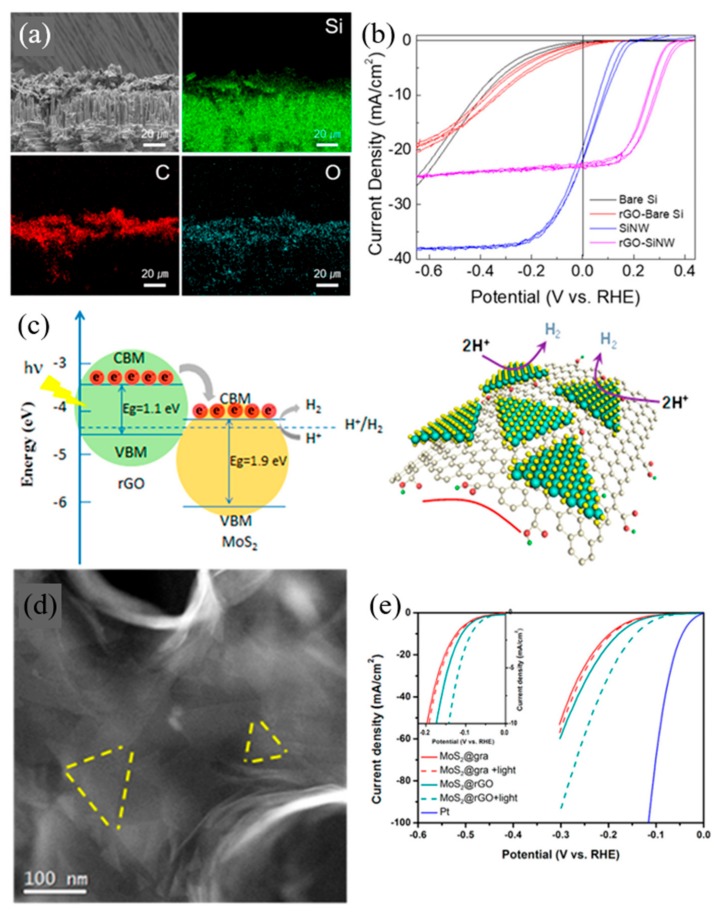
Reduced graphene oxide (rGO) applied in photoelectrochemical cells (**a**,**b**), as photocatalysts (**c**), and electrochemical cells (**d**,**e**) for water splitting reactions. (**a**) Cross-Sectional SEM image of rGO on silicon nanowire (SiNW) photoelectrode and corresponding EDS elemental mapping describing the presence of Si, C and O, (**b**) Photoelectrochemical responses for bare Si, SiNW, rGO-bare Si and rGO-SiNW. Different lines of the same color represent repeated measurements at the same condition [7], (**c**) Schematic illustration of band alignment and mechanism of photo-excited charge transfer in MoS_2_/rGO (left) and Schematic image of MoS_2_/rGO, (**d**) HAADF-STEM image showing the morphology of MoS_2_/rGO, (**e**) Polarization curves with/without light irradiation [49].

Sim et al. [7] investigated the utilization of reduced graphene oxide as a cocatalyst with silicon nanowire to enhance the PEC response towards HER. SiNW has gained significant attention in the area of photon-assisted light-harvesting, owing to its narrow bandgap and its promising photovoltaic applicability. However, due to the high overpotential required for hydrogen generation, the use of the silicon electrode is highly challenging. rGO, with its exceptional PEC water splitting capability and fast-interfacial charge transfer, is a suitable co-catalyst. rGO displayed remarkable catalytic activity towards PEC HER on SiNW photocathodes, as shown in Figure 4b, with a solar-to-hydrogen (STH) efficiency rate 3.47 times higher than bare SiNW. The overpotential of rGO-SiNW measured 0.239 V vs. RHE at a current density of −10 mA·cm^−2^ and was calculated to 3.16% of half solar-to-hydrogen conversion efficiency whereas the bare SiNW was measured at 0.09 V overpotential and 0.91% half STH. The onset potential at −1 mA·cm^−2^ exhibited a positive shift from 0.179 V of SiNW to 0.326 V of rGO-SiNW, thereby proving an enhancement of PEC HER.

Zhang et al. [49] have worked with MoS_2_ and rGO heterojunctions for photo-enhanced HER, as shown in Figure 4c,d. In their work, the nanoporous heterojunctions were comprised of 3D nanoporous rGO as the light absorber and a 2D monolayer of MoS_2_ as the electron transfer bridge and the electrocatalyst for PEC hydrogen production, respectively. High electric conductivity and high photoresponsivity of rGO showed a synergetic effect with MoS_2_, the HER electrocatalyst. Also, utilizing the tunablity and gaps of rGO, is advantageous in generating a built-in electric field so as to suppress electron-hole recombination and to promote the catalytic activity of MoS_2_. Compared to obvious photo-enhancement with rGO, MoS_2_ with graphene showed a minor light response, evidence that rGO plays a crucial role in the PEC system of HER catalysis. With graphene, overpotentials at a current density of −10 mA·cm^−2^ were 197 mV under dark and 193 mV under one-sun illumination resulting in 4 mV of photo-enhancement. MoS_2_/rGO resulted in 32 mV photo-enhancement with an overpotential of 173 mV under dark, and 141 mV under illumination at −10 mA·cm^−2^ current density, which is eight times the enhancement of MoS_2_/graphene, as shown in Figure 4e.

Inorganic semiconductor photoelectrodes composed of transition metal dichalcogenides (oxides, sulfides, and selenides) such as ZnS [50], ZnO [51], Fe_2_O_3_ [52,53], have been investigated with the rGO as a cocatalyst. Even with large bandgaps these semiconductors have fast-interfacial charge transferring properties of rGO thus enhancing performance of solar-driven water splitting reactions. Hou et al. [54] have investigated the role of rGO in PEC water splitting reactions. α-Fe_2_O_3_/rGO/BiV_1−x_Mo_x_O_4_ Core-Shell heterojunction arrays were synthesized with and without rGO. A BiV_1−x_Mo_x_O_4_ photocatalyst absorbed the visible light thereby generating the electron-hole pair. Assisted by rGO, these electrons are then transferred through ferrite to the cathode. Due to the conductive properties of rGO, the electrons are efficiently transferred so as to inhibit the recombination of photogenerated electron-hole pairs. This mechanism was also shown with other PEC systems.

Similarly, Ning et al. [55] have investigated rGO with TiO_2_/NiFe-layered double hydroxide (NiFe-LDH) composite for enhanced PEC performance. To begin with, TiO_2_, under illumination, generates the electron-hole pairs. Following this, owing to the band energy difference and chemical binding interactions between the TiO_2_/rGO/NiFe-LDH components, the rapid migration of electrons to the current collector occurred due to the superior electron mobility exhibited by rGO. Simultaneously, the holes are collected to NiFe-LDH along with the oxidation of Fe, which acts as the active site for water oxidation. With the support of rGO, the PEC was greatly enhanced as compared to TiO_2_/NiFe-LDH composite and TiO_2_ with rGO only. These works have shown the role of conductive rGO in PEC water splitting reactions. The pristine TiO_2_ nanoarrays displayed 0.92 mA·cm^−2^ photocurrent density at 0.6 V (vs. SCE) which is relatively low compared to 1.18 mA·cm^−2^ of TiO_2_/NiFe-LDH, 1.5 mA·cm^−2^ of TiO_2_/rGO, and 1.74 mA·cm^−2^ of the ternary TiO_2_/rGO/NiFe-LDH nanoarrays. An overall 1.89-fold enhancement of photocurrent density was observed. The increment of photocurrent density between TiO_2_/LDH and TiO_2_/rGO/LDH can be indicated to highlight the role of rGO in this ternary nanoarray structure. 1.74 mA·cm^−2^ photocurrent density of TiO_2_/rGO/LDH is 1.47-fold higher than 1.18 mA·cm^−2^ of TiO_2_/LDH. This can also be noticed through the comparison between the TiO_2_/rGO and bare TiO_2_. TiO_2_/rGO has exhibited 1.63 times greater photocurrent density than bare TiO_2_ nanoarrays. Similar results have been shown using other metal oxide semiconductors such as α-Fe_2_O_3_ and WO_3_ with rGO/NiFe-LDH decorations. The improved charge transport, arising from rGO, allows for better separation of the electron-hole pair to suppress surface recombination thereby enhancing PEC systems.

### 2.3. Graphitic Carbon Nitride

Graphitic carbon nitride (g-C_3_N_4_) is regarded as a good photocatalyst for solar fuel conversion and pollutant degradation due to its moderate bandgap of 2.7 eV, good chemical stability under light irradiation, low cost and nontoxicity [56,57]. The pyridinic and quaternary nitrogen atoms act as active sites for chemical reactions, making g-C_3_N_4_ a favorable catalyst in water splitting reactions [36]. Considerable work has been done to optimize its performance, such as doping, forming heterojunctions [58,59], metal nanoparticle decoration, and copolymerization. A g-C_3_N_4_ powder can be easily synthesized via inexpensive raw materials, such as urea, melamine, dicyandiamide, under high temperatures, further adding to its advantages as a catalyst in water splitting reactions [23,24]. Through techniques, such as spin coating, drop-casting, and the doctor blade method, g-C_3_N_4_ powder can be deposited onto solid substrates in PEC water splitting systems [60].

Peng et al. [61] adopted the doctor blade method for the synthesis of porous carbon nitride films on a fluorine-doped tin oxide (FTO) substrate, which showed a high of 13 μA·cm^−2^ at 1.23 V vs. RHE, which decreased to 60% of the initial photocurrent density after 2 h during long-term measurement. Mohamed et al. [62] synthesized g-C_3_N_4_ through thermal polymerization from urea. This was then deposited onto the FTO substrate using the spin coating method. The highest photocurrent density of approximately 21 μA·cm^−2^ was obtained in this study at 1.23 V vs. Ag/AgCl in 0.5M Na_2_SO_4_ electrolyte. Spray coating on to FTO glass, another conventional method, was carried out by Sima et al. [63] The group synthesized g-C_3_N_4_ by pyrolysis of urea. This was then dispersed in methanol to attain exfoliated nanosheets of g-C_3_N_4_ for spray coating. As deposition is time dependent, 3 μA·cm^−2^ of photocurrent density was obtained at 1.23 V vs. RHE when the FTO was sprayed for 270 s. As mentioned above, doctor blade methods and spin coating methods are simple and straightforward. However, the low levels of uniformity of g-C_3_N_4_ films obtained through these methods has limited the applications of g-C_3_N_4_ in PEC systems. As a result, other experiments into obtaining a uniform coating with even coverage over the substrate have been researched. For example, thermal deposition techniques have been found to produce films with higher uniformity and coverage on the substrates.

Bian et al. [64] have introduced a thermal vapor condensation method for the deposition of g-C_3_N_4_ on a large area of different substrates. Through heating precursors, such as melamine, dicyandiamide, to a temperature greater than 300 °C, precursor vapor is generated in the crucible. Condensation of the vapor can easily deposit g-C_3_N_4_ onto the surface of the substrate, which is positioned at the top of the crucible. The g-C_3_N_4_ was also grown on FTO glass as well as ITO glass, silica, and glass. Even though the morphologies on the glass substrate were less uniform, the deposition on FTO, ITO, and silica substrates was successfully grown uniformly. The group also reported that using dicyandiamide, thiourea, and urea could not achieve a g-C_3_N_4_ film with as much uniformity as using melamine as the precursor. The g-C_3_N_4_ film grown on FTO glass, processed at 600 °C showed the best photoresponsivity with an onset potential of 0.35 V vs. RHE. The photocurrent density of 0.12 mA·cm^−2^ at 1.55 V vs. RHE was also achieved with Na_2_S as the sacrificial reagent.

**Figure 5:**
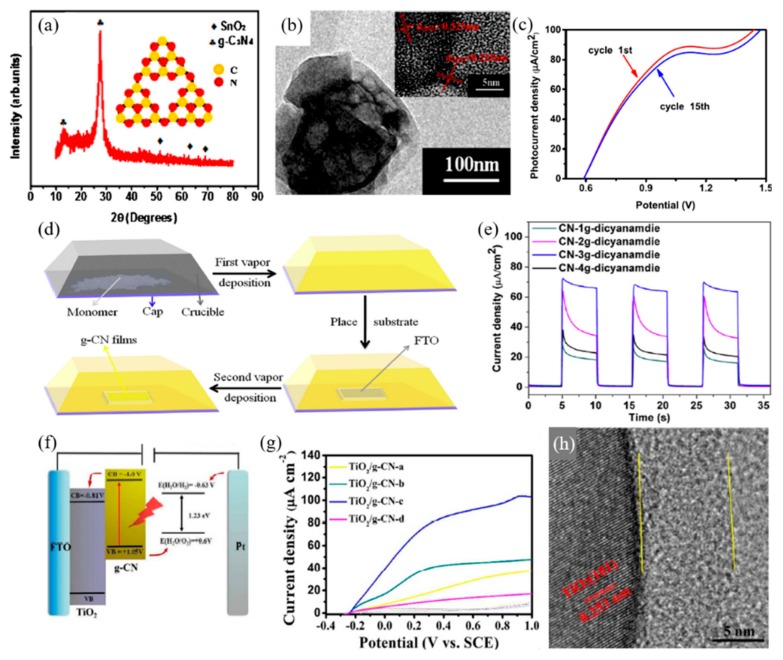
Graphitic carbon nitride (g-C_3_N_4_) applied in photoelectrochemical cells for water splitting reactions. (**a**) XRD of the framework g-C_3_N_4_ film on FTO, inset shows the tri-s-triazine units of g-C_3_N_4_, (**b**) TEM image of the framework g-C_3_N_4_, inset shows the HRTEM image, (**c**) Linear sweep voltammograms (LSV) curve of the framework g-C_3_N_4_ film [65]. (**d**) Schematic diagram of the thermal vapor deposition (TVD) procedure for deposition of g-C_3_N_4_ films on FTO. (**e**) Transient photocurrent density under light illumination of the g-C_3_N_4_ films prepared using different amount of dicyanamide [23]. (**f**) Schematic diagram of PEC water oxidation mechanism on the TiO_2_/g-C_3_N_4_ arrays under visible light, (**g**) Linear sweep voltammograms (LSV) of TiO_2_/g-C_3_N_4_ Core-shell Arrays under illumination (λ > 420 nm) and dark states. (**h**) HRTEM image of TiO_2_/g-C_3_N_4_ composite [66].

Lu et al. [65] reported a thermal vapor liquid-polymerization for the framework g-C_3_N_4_ film on the FTO glass substrate directly. Through the framework film, the PEC performance of g-C_3_N_4_ was improved due to its uniform surface, increased light-harvesting ability, optical performance, and reduced recombination of photogenerated EHP. It has been indicated that using the framework structure enhances the light-harvesting ability of g-C_3_N_4_ by increasing the reflections of light as well as enhancing the photogenerated charge separation due to the faster charge transfer rate. The photocurrent density of the framework g-C_3_N_4_ film, shown in Figure 5c, reached 89 μA·cm^−2^, which is twice the current density of 40 μA·cm^−2^ in dark conditions.

Similarly, Lv et al. [23] reported a two-step thermal vapor deposition process of depositing g-C_3_N_4_ films. As shown in Figure 5d, the film was firstly deposited onto the inner wall of the crucible through the first TVD method, following this the g-C_3_N_4_ attached to the crucible was vaporized through the second process, allowing it to grow on the substrate. The deposition of the g-C_3_N_4_ film was determined by temperature, where the film failed to grow on the substrate below 450 °C and fully detached above 580 °C. The optimal deposition temperature was set selected at approximately 500 °C. As for the PEC properties, the quantity of precursor showed an important effect. In Figure 5e, the quantity of dicyanamide was controlled from 1 g to 4 g, with PEC performance improving as the quantity increases. However, the use of 4 g of the monomer exhibited a sharp decrease in photocurrent density where dicyanamide was used as the precursor. This two-step method exhibited the highest photocurrent density compared to melamine and cyanamide, with 63, 52 and 39 μA·cm^−2^, respectively. This two-step TVD deposition strategy can be employed in the synthesis of heterojunctions for the further enhancement of PEC performance.

The g-C_3_N_4_ electrodes, synthesized directly to the substrates through the thermal vapor deposition method, shows higher photocurrent density overall compared to one that has been deposited through conventional doctor blade or spin/spray coating methods. However, it should be noted that for mass production more straight forward methods are preferable as mass production through thermal vapor deposition methods is limited. Studies of g-C_3_N_4_ in forming heterojunctions with other photoelectrodes have been developed so as to enhance the efficiency of the PEC system, compensating for the loss of simplicity in the synthesis process [59]. The excellent conductive properties of g-C_3_N_4_, allowing for the formation of heterojunctions with other semiconductors, enhances charge separation and increases the water splitting reactions. Fan et al. [66] fabricated TiO_2_/g-C_3_N_4_ core-shell arrays to investigate the synergetic effects between TiO_2_ and g-C_3_N_4_ for the PEC water splitting system. Firstly, TiO_2_ arrays were grown on FTO glass through the hydrothermal process, then decorations of g-C_3_N_4_ layers were grown on top of TiO_2_ through a solvothermal process. The formation of heterojunctions between TiO_2_ and g-C_3_N_4_, shown in Figure 5f, allows for the efficient transfer and separation of photogenerated charge carriers for the enhancement of PEC systems. The growth of TiO_2_ and the quantity of g-C_3_N_4_ were studied as both can be a factor in the PEC system. With g-C_3_N_4_, the TiO_2_ arrays grown for 4 h showed the highest photocurrent density value of 43 μA·cm^−2^ at 0.6 V vs. SCE, which is 19.7 times higher than bare TiO_2_, and 11.7 times higher than pure g-C_3_N_4_. The quantity of g-C_3_N_4_ was controlled by the quantity of precursor used. As the amount of the precursor increases, the PEC performance tends to decrease due to the reverse recombination of photogenerated charge carriers within the overgrown g-C_3_N_4_. The addition of 1 mmol of cyanuric acid and 0.5 mmol of melamine in the deposition of g-C_3_N_4_ on top of TiO_2_, resulted in the highest PEC performance observed at 81 μA·cm^−2^, which is 36 times higher than that of pure TiO_2_.

A similar concept of heterojunctions of TiO_2_ and g-C_3_N_4_ has been raised by Liu et al. [58] through the anodizing of titanium foil to form TiO_2_ nanotube arrays (TNTAs) then decorating with g-C_3_N_4_. For comparison, amorphous TiO_2_, Ti foil without anodic oxidation treatment was also investigated. The photocurrent density of g-C_3_N_4_ decorated TNTAs (g-C_3_N_4_/TNTA) was about 0.86 mA·cm^−2^ at 1.23 V vs. RHE while pristine TNTAs only measured approximately 0.40 mA·cm^−2^ at 1.23 V. The photocurrent densities of g-C_3_N_4_decorated TiO_2_ and pristine TiO_2_ were determined to be 0.19 and 0.07 mA·cm^−2^, respectively. The lower current density of TiO_2_, compared to TNTAs, refers to the distinct photo-response of TNTAs. However, in both cases of g-C_3_N_4_/TNTAs and g-C_3_N_4_/TiO_2_, an increase in photocurrent density occurred with g-C_3_N_4_ decoration. The photocurrent density of g-C_3_N_4_/TNTAs was twice the density of pristine TNTA, while amorphous TiO_2_ increased to a 2.7-fold higher photocurrent density with the g-C_3_N_4_ decorations.

The high content of nitrogen in carbon material has facilitated high reactivity during the chemical reactions. g-C_3_N_4_ has also shown excellent performance in EC water splitting. Ma et al. [67] have conducted studies on a g-C_3_N_4_/Carbon Nanotube (CNT) three-dimensional (3D) composite for OER electrocatalysts. The 2D layer of g-C_3_N_4_ showed outstanding activity for OER originating from its high nitrogen contents, along with its unique porous structure with CNT. The composite showed an operating potential of 1.60 V vs. RHE to achieve the current density of 10 mA·cm^−2^ with a Tafel value of 83 mV·dec^−1^, which is a similar value to that of 1.59 V vs. RHE and 90 mV·dec^−1^ for IrO_2_-CNT, one of the noble-metal catalysts. The composite with 2D g-C_3_N_4_ showed improved OER performance compared to that with bulk g-C_3_N_4_, owing to an increase in surface area in the 2D structure of g-C_3_N_4_. Additionally, it can be noted that comparatively poor electrical conductivity is compensated through the smooth current transfer from the π–π interaction of the two carbon-based materials.

Sulfur-modified g-C_3_N_4_, reported by Kale et al. [3], has shown excellent catalytic properties due to minimization of the activation energy for the OER. Inspired by melamine nanogeodes (MNG), the group synthesized the g-C_3_N_4_ nanostructure from MNG powder, which was obtained through a simple hydrothermal process with melamine. Geodes are hollow, circular volcanic rocks formed in nature. The multiwalled closed cage structure of nano-sized geodes of melamine is mixed with a sulfur precursor which then undergoes a two-step heating process in order to obtain S-modified g-C_3_N_4_. The overpotential of 290 mV achieved with this structure is lower than that of the novel-metal as well as that of non-metal, metal/metal oxide catalysts.

### 2.4. Graphene Quantum Dots/Graphene Quantum Sheets

Graphene quantum dots (GQDs) are zero-dimensional (0D) derivatives of graphene with a diameter of up to ~20 nm [25]. Due to quantum confinement and edge effects, GQDs possess a distinct bandgap and increment of surface area [68]. They possess characteristics such as low-toxicity, chemical inertness, and biocompatibility, facilitating their application in many fields, especially those of photoelectronics and catalysis [69]. Additionally, GQD possesses a high degree of crystallinity relating to superior electron-transport capability and an enhanced carrier lifetime [70,71,72].

**Figure 6:**
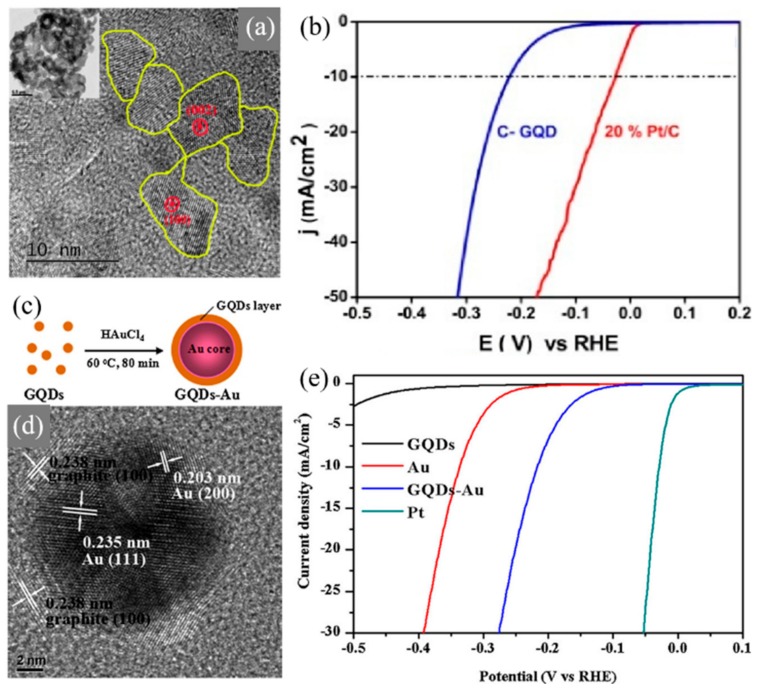
Graphene quantum dots (GQDs) applied in electrochemical cells for water splitting reactions. (**a**) HRTEM image of inter-connected graphene quantum dots (c-GQDs), inset shows low magnification image of c-GQDs. (**b**) Polarization curve for 20% Pt/C and c-GQDs [73]. (**c**) Schematic illustration of the strategy for GQDs-Au preparation. (**d**) TEM image of single GQDs-Au. (**e**) Polarization curves obtained with several catalysts as indicated [74].

The electrocatalytic performance of nitrogen functionalized inter-connected GQDs (c-GQDs) was reported by Kundu et al. [73] GQDs were treated with hydrazine hydrate in acidic conditions so as to synthesize 3D c-GQDs. 0D GQDs are inter-connected through a form of hydrazine bonding, as shown in Figure 6a. The c-GQDs exhibited a smaller overpotential of 220 mV with 20% Pt/C was benchmarked at 50 mV, as shown in Figure 6b. The c-GQDs also exhibited excellent durability even after 1000 cycles. The results of synthesizing c-GQDs shows that it is a promising candidate for hydrogen production and opens new prospects for carbon-based catalysts. Lou et al. [74] reported on graphene quantum dots/gold (Au) hybrid nanoparticles as an electrocatalyst for HER, as shown in Figure 6c,d. Au nanoparticles are known for their high catalytic activity supporting metal oxide catalysts, as well as carbon-based catalysts, including GQDs. The hybrid nanoparticles showed enhanced electrochemical performance towards HER, compared to pure GQDs and pure Au nanoparticles. The Tafel value of both GQDs/Au and pure Au nanoparticle is approximately equal at 75–78 mV·dec^−1^. However, as shown in Figure 6e, the overpotential of 0.14 V was achieved with GQDs/Au hybrid nanoparticles for HER, whereas pure Au nanoparticles exhibit a higher overpotential of 0.27 V, indicating that hybrid nanoparticles exhibit significantly higher electrocatalytic activity than pure Au.

**Figure 7:**
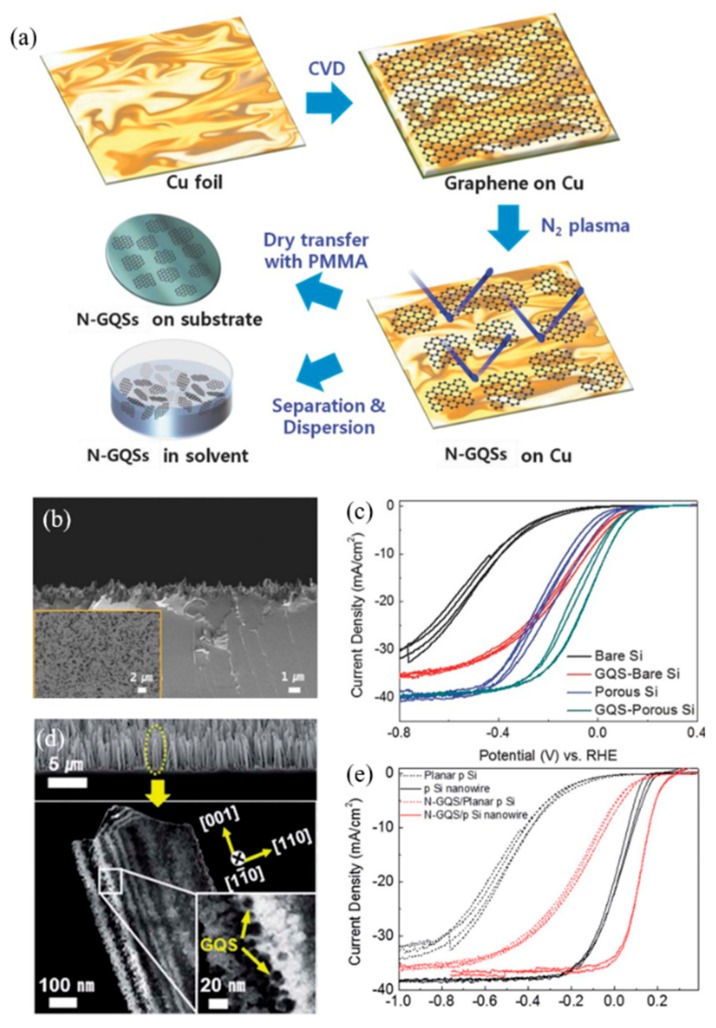
Graphene quantum sheets (GQSs) applied in photoelectrochemical cells for water splitting reactions. (**a**) Schematic illustration of N-doped graphene quantum sheets (N-GQSs) fabrication processes. (**b**) Cross-section SEM image of porous Si, inset shows top-view SEM image of porous Si. (**c**) Cyclic Voltammetry (CV) of N-GQSs on bare Si and porous Si [27]. (**d**) Cross-section SEM image and dark-field TEM images of N-GQSs on p-type silicon nanowires (p-SiNWs). (**e**) Photocurrent density—potential (J–E) curves for lightly boron-doped planar p-Si electrode and silicon nanowire electrode deposited with N-GQSs [28].

Atom-thick Graphene Quantum Sheets (GQSs) were introduced into the PEC system as a co-catalyst for the water splitting system [75]. Moon et al. [27] introduced nitrogen-doped graphene quantum sheets (N-GQSs) for solar-driven hydrogen evolution, as shown in Figure 7. N-GQSs can be synthesized through a solvent-free method, which is highly advantageous compared to the complicated wet-chemical reactions required for synthesis of GQDs. By applying nitrogen plasma to as-grown graphene on Cu, the sheet of graphene can be converted to N-GQSs. The resulting N-GQSs can be transferred as a film-like layer or easily dispersed in an organic solvent to be transferred onto a Si substrate as a PEC catalyst for HER. Sim et al. [28] applied the N-GQSs to both planar Si as well as a porous Si photoelectrode. By inducing N-GQSs, the onset potential was positively shifted by 0.29 V for planar Si, and 0.09 V for porous Si. With a potential of −5 mA·cm^−2^ similar values of 0.3 V and 0.11 V, planar Si and porous Si, respectively, were observed. The positive shift of the overall J–E curves induced by the N-GQSs indicates an enhancement of HER performance. The group further experimented with N-GQSs on Si nanowire (SiNW). Similarly, an enhanced HER performance can be observed with a positive shift of 0.09 V for the onset potential, in addition to a high ABPE of 2.29%, which is 2.5-fold higher than that of bare SiNW.

**Figure 8:**
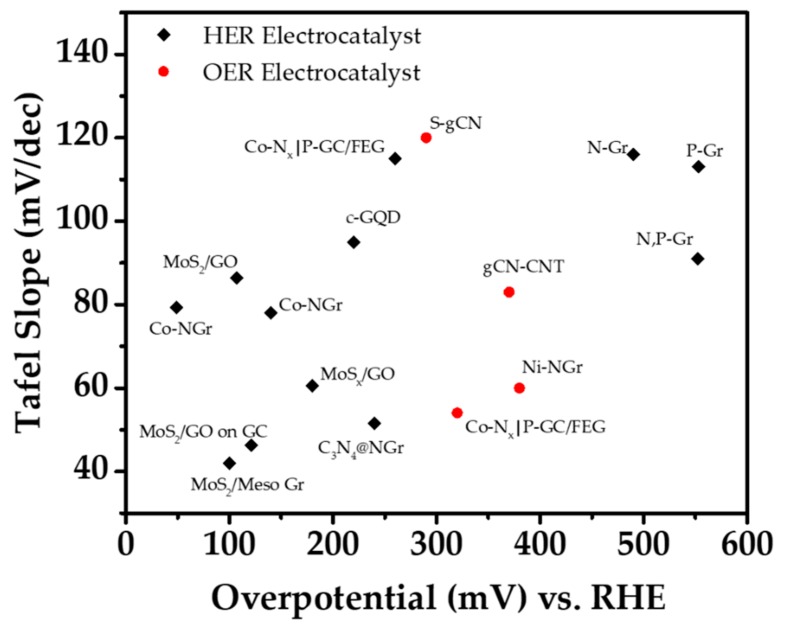
Plot for carbon-based materials in electrochemical systems.

We went through the low-dimensional carbon-based materials for the water splitting reactions. The materials are summarized below for electrochemical, photocatalysis, and photoelectrochemical system. The few key parameters measured for electrochemical systems are the Tafel slope and the overpotential to investigate the performance of the materials. Figure 8 shows a simple plot for the low-dimensional carbon-based materials in EC water splitting along with the detailed information and references in Table 1. As for the photocatalysis which is shown in Table 2, the H_2_ production rate and the light source are the important factor. Similar with photocatalysis, the intensity of the light source is a crucial factor in photoelectrochemical system. The onset potential measured when the current density reaches −1 mA·cm^−2^, and the overpotential, which is measured when the current density reaches −10 mA·cm^−2^, are the important factors in PEC hydrogen evolution along with the light intensity. However, in some cases where the materials show relatively low current density, the photocurrent density is measured to evaluate the performances. Likewise, the current density of the oxygen evolution performances is relatively low compared to HER, therefore the photocurrent density is measured along with the light intensity. The HER performances are shown in Table 3 while Table 4 shows the OER performances of the low-dimensional carbon-based materials.

## 3. Conclusions

As the search for environment-friendly energy source progresses, hydrogen produced from water splitting reactions is proving to be a possible candidate. However, efficient energy conversion in water splitting reactions requires further optimization through the development of suitable catalysts. Novel-metal and numerous semiconductor-based materials are available for the enhancement of catalytic performance. However, low cost, non-toxic, abundant, and highly efficient catalysts are vital for their commercial use. Due to their unique electrical and catalytic properties, carbon materials exhibit outstanding performance as both catalyst and cocatalyst and are promising candidates for worldwide commercial use. Because of its layered 2D structure and π–conjugated planes, graphene exhibits excellent electrical properties, enhancing the separation of photogenerated EHPs and electron transfer for catalytic reactions. Furthermore, the transparent characteristic of graphene, makes it suitable as a cocatalyst also. The oxygenated functional groups of the GO and rGO act as active sites for the water splitting reaction with defects of the functional groups opening the bandgap thereby enabling the absorption of light energy. With the created bandgap, GO and rGO is also suitable for creating heterojunctions with other carbon materials as well as the semiconductor-based catalyst thereby improving charge separation and reducing recombination probability. The pyridinic and quaternary nitrogen atoms in g-C_3_N_4_ also act as reaction sites, similar to oxygenated functional groups in GO and rGO. The simple synthesis methods and high abundancy of these 2D materials are advantageous aspects of the multi-purpose catalyst for use in water splitting reactions. Furthermore, 0D GQDs and GQSs have also shown outstanding performance as catalysts. Due to the quantum confinement effects of these 0D materials, GQDs and GQSs exhibit increased surface area and distinct bandgaps, making them favorable catalysts.

In summary, low-dimensional carbon-based materials are being widely studied for the enhancement of photo and electrochemical water splitting reactions, as well as photocatalysis. These materials have been found to exhibit suitable characteristics for use as catalysts due to their exceptional performance and ecofriendly properties. Many derivatives of the carbon materials are available, and they have been co-opted for multiple uses. Numerous combinations of other catalysts, as well as other derivatives, are available due to the flexible optimization of the carbon materials. Low-dimensional carbon materials exhibit enormous potential as highly efficient, low cost and ecofriendly catalysts in water splitting reactions for the production of hydrogen as an energy source.

## Figures and Tables

**Table 1:** Carbon-based materials for electrochemical water splitting reactions.

Reaction	Material	Overpotential (mV vs. RHE)	Tafel Slope (mV/dec)	Electrolyte	Ref.
HER	N-Gr	490	116	0.5M H_2_SO_4_	[76]
	P-Gr	553	113		
	N, P-Gr	552	91		
	Co-NGR	49	79.3	0.5M H_2_SO_4_	[44]
	C_3_N_4_@NG	240	51.5	0.5M H_2_SO_4_	[77]
	MoS_2_/Meso graphene	100	42	0.5M H_2_SO_4_	[78]
	Co-N_x_|P-GC/FEG	260	115	1M KOH	[37]
	MoS_x_/GO	180	60.5	0.5M H_2_SO_4_	[47]
	MoS_2_/GO	107	86.3	0.5M H_2_SO_4_	[46]
	MoS_2_/GO on Glassy Carbon	121	46.3	0.5M H_2_SO_4_	
	c-GQD	220	95	0.5M H_2_SO_4_	[73]
	GQD/Au	140	78	0.5M H_2_SO_4_	[74]
OER	Co-Nx|P-GC/FEG	320	54	1M KOH	[37]
	Ni-NGR	380	60	1M KOH	[43]
	S-g-C_3_N_4_	290	120	1M (KOH + NaClO_4_)	[3]
	g-C_3_N_4_/CNT	370	83	0.1M KOH	[67]

**Table 2:** Carbon-based photocatalysts for hydrogen production.

Material	H_2_ Generation Rate	Electrolyte	Light Source	Ref.
Cu/Gr/TiO_2_	63.75 mmoL/g·h	0.1M NaClO_4_ + 10 vol.% methanol	300 W Hg Lamp	[40]
NGr/TiO_2_	13.72 μmoL/h	TEOA 10 vol.%	150W Xe Lamp	[42]
MoS_2_/Graphene-TiO_2_	1989 μmoL/g·h	20 vol.% methanol	300W Xe Lamp 545 mW/cm^2^	[79]
GO	5.67 mmoL/g·h	20 vol.% methanol	400W Hg Lamp	[12]
TiO_2_/B-g-C_3_N_4_	150 μmoL/g·h	20 vol.% methanol	300 W Xe Lamp 420 nm filter	[36]
NiS/Ag/g-C_3_N_4_	9.728 mmoL/g·h	TEOA 10 vol.%	300 W Xe Lamp 46.31 mW/cm^2^	[80]
P-TCN/GQDs	112.1 μmoL/h	20 vol.% methanol	300 W Xe Lamp 420 nm filter	[26]
N-GO QD	0.45 μmol/g h	Water	300 W Xe Lamp 420 nm < λ < 800 nm	[68]
NGQDs-Cu_2_O	22.6 μmol/g h	20 vol.% methanol	300 W Xe Lamp 420 nm filter	[81]

**Table 3:** Carbon-based materials for photoelectrochemical HER.

**Material**	**Onset Potential** **(@ −1 mA/cm^2^)** **V vs. RHE**	**Over Potential** **(@ −10 mA/cm^2^)** **V vs. RHE**	**Electrolyte**	**Light Source**	**Ref.**
Gr-Si	0.01	−0.21	1M HClO_4_	300W Xe Lamp AM1.5 100 mW/cm^2^	[38]
NGr-Si	0.12	−0.04			
Pt-NGr-Si	0.35	0.25			
double-layer Gr-Si	0.05	−0.11	1M HClO_4_	300W Xe Lamp AM1.5 100 mW/cm^2^	[39]
Plasma Double-layer Gr-Si	0.15	0.01			
SiNW/rGO	0.08 *	−0.13 *	H_2_SO_4_ + 0.5M K_2_SO_4_	300W Xe Lamp 100 mW/cm^2^	[82]
MoS_2_/rGO	−0.048 *	−0.141 *	0.5M H_2_SO_4_	AM1.5 100 mW/cm^2^	[49]
rGO-SiNW	0.326	0.239	1M HClO_4_	100W Xe Lamp AM1.5 100 mW/cm^2^	[7]
GQS-bare Si	0.12	0.01 (@ −5mA/cm^2^)	1M HClO_4_	300W Xe Lamp AM1.5 100 mW/cm^2^	[27]
GQS-porous Si	0.16	0.08 (@ −5mA/cm^2^)	1M HClO_4_		
N-GQSs/planar Si	0.13	−0.04	1M HClO_4_	300W Xe Lamp AM1.5 100 mW/cm^2^	[28]
N-GQSs/ SiNW	0.26	0.16	1M HClO_4_		
**Material**	**Photocurrent Density** **(mA/cm^2^)**	**Measured Potential**	**Electrolyte**	**Light Source**	**Ref.**
Cu-CN-W	200 μA/cm^2^	0.42 V vs. RHE	0.2M Na_2_SO_4_	300W Xe Lamp AM1.5 420 nm filter	[57]
Gr/Cu_2_O/Cu mesh	4.8	0 V vs. RHE	1M Na_2_SO_4_ + 0.1M Potassium Phosphate	AM1.5 100 mW/cm^2^	[83]
CuBi_2_O_4_/rGO	0.94	0 V vs. RHE	0.5M Na_2_SO_4_	300W Halogen Lamp 100 mW/cm^2^	[84]
rGO/Cu_2_O/Cu foil	2.3	0 V vs. RHE	0.5M Na_2_SO_4_	50W Halogen Tungsten Lamp 85 mW/cm^2^	[85]

* indicates the values measured and extrapolated by our group referring to the figures and data from other paper.

**Table 4:** Carbon-based materials for photoelectrochemical OER.

Material	Photocurrent Density (mA/cm^2^)	Measured Potential	Electrolyte	Light Source	Ref.
g-C_3_N_4_	89 μA/cm^2^	1.1 V vs. RHE	0.1M Na_2_SO_4_	Xe Lamp AM1.5 100 mW/cm^2^	[65]
g-C_3_N_4_	0.12	1.55 V vs. RHE	0.1M Na_2_SO_4_ + 0.01M Na_2_S	Xe lamp AM1.5 100 mW/cm^2^	[64]
g-C_3_N_4_, dicyanamide	63 μA/cm^2^	1.23 V vs. RHE	0.1M Na_2_SO_4_	300W Xe Lamp AM1.5 100 mW/cm^2^	[23]
g-C_3_N_4_, melamine	52 μA/cm^2^				
g-C_3_N_4_, cyanamide	39 μA/cm^2^				
g-C_3_N_4_	20.73 μA/cm^2^	1.23 V vs. Ag/AgCl	0.5M Na_2_SO_4_	Xe Lamp 100 mW/cm^2^	[62]
g-C_3_N_4_	45 μA/cm^2^	0.86 V vs. RHE	0.2M Na_2_SO_4_	500 W Xe Lamp 420 nm filter	[60]
g-C_3_N_4_/NiCo-LDH	11.8 μA/cm^2^	0.6 V vs. SCE	0.2M Na_2_SO_4_	200 W Xe lamp 100 mW/cm^2^ 420 nm filter	[59]
TiO_2_/g-C_3_N_4_	3.6 μA/cm^2^	1.23 V vs. RHE	0.5M Na_2_SO_4_	300 W Xe Lamp AM1.5 100 mW/cm^2^	[63]
TiO_2_/g-C_3_N_4_ Core Shell array	80.9 μA/cm^2^	0.6 V vs. SCE	0.2M Na_2_SO_4_	200 W Xe Lamp 20 mW/cm^2^ 420 nm filter	[66]
TiO_2_-CN	29.4 μA/cm^2^	1.23 V vs. RHE	0.5M Na_2_SO_4_	300W Xe Lamp AM 1.5 420 nm filter	[86]
TiO_2_@ g-C_3_N_4_@CoPi	1.6	1.23 V vs. RHE	0.1M Na_2_SO_4_	300W Xe Lamp AM1.5 100 mW/cm^2^	[87]
g-C_3_N_4_/BiOI	0.7	0.8 V vs. Ag/AgCl	0.1M Na_2_SO_4_	AM1.5 100 mW/cm^2^	[88]
g-C_3_N_4_/TNTA	0.86	1.23 V vs. RHE	0.1M Na_2_SO_4_	300W Xe Lamp AM1.5 100 mW/cm^2^	[58]
Co-N_x_|P-GC/FEG/Fe_2_O_3_	2.15	1.23 V vs. RHE	1M KOH	AM1.5 100 mW/cm^2^	[37]
CN/rGO	72 μA/cm^2^	1.23 V vs. RHE	0.1M KOH	AM1.5 100 mW/cm^2^	[89]
	660 μA/cm^2^	1.23 V vs. RHE	0.1M KOH + 10 vol.% TEOA		
TiO_2_/rGO/NiFe-LDH	1.74	0.6 V vs. SCE	0.5M Na_2_SO_4_	150W Xe Lamp 100 mW/cm^2^	[55]
Fe_2_O_3_-rGO	1.06	1.23 V vs. RHE	1M NaOH	300W Xe Lamp AM1.5 100 mW/cm^2^	[53]
BVO/rGO	554.44 μA/cm^2^	1.2 V vs. Ag/AgCl	0.1M Na_2_SO_4_	300W Xe Lamp AM1.5 100 mW/cm^2^	[90]
rGO/γ-Fe_2_O_3_	6.74	1.8 V vs. RHE	1M NaOH	360 nm UV light	[91]
α-Fe_2_O_3_/Gr/BiV_1_−____x__Mo_x_O_4_	1.97	1.0 V vs. Ag/AgCl	0.01M Na_2_SO_4_	150W Xe Lamp AM1.5 64 mW/cm^2^ 420 nm filter	[54]
Au_x_/GQDs/NP-TNTAs	1.1 *	0 V vs. Ag/AgCl	0.5M Na_2_SO_4_	300 W Xe Lamp AM1.5 100 mW/cm^2^	[71]
GQD@ZnO	0.34	0.6 V vs. Pt	0.5M Na_2_SO_4_	150 W Xe Lamp 100 mW/cm^2^	[69]
N-GQD-ZnO	2.45	1 V vs. Ag/AgCl	0.1M Na_2_SO_4_	270W Xe Lamp	[92]

* indicates the values measured and extrapolated by our group referring to the figures and data from other paper.

## Abbreviations

List of abbreviations mentioned in the main text.

0D	Zero-Dimensional
2D	Two-Dimensional
3D	Three-Dimensional
BVO	BiVO_4_
CB	Conduction Band
c-GQD	Inter-Connected Graphene Quantum Dots
CN	Carbon Nitride
CNT	Carbon Nanotube
CV	Cyclic Voltammetry
EC	Electrochemistry/Electrochemical
EDS	Energy Dispersive X-ray Spectroscopy
EHP	Electron-Hole Pair
FTO	Fluorine-doped Tin Oxide
g-C3N4	Graphitic Carbon Nitride
GO	Graphene Oxide
GO QD	Graphene Oxide Quantum Dot
GQD	Graphene Quantum Dot
GQS	Graphene Quantum Sheet
Gr	Graphene
HAADF-STEM	High-angle Annular Dark-field Scanning Transmission Electron Microscopy
HER	Hydrogen Evolution Reaction
HRTEM	High-resolution Transmission Electron Microscopy
ITO	Indium-doped Tin Oxide
LDH	Layered Double Hydroxide
LSV	Linear Sweep Voltammetry
MNG	Melamine Nanogeodes
N-GQD	N-doped Graphene Quantum Dot
N-GQS	N-doped Graphene Quantum Sheet
NGR	N-doped Graphene
NP	Nanoparticle
OER	Oxygen Evolution Reaction
PEC	Photoelectrochemisty / Photoelectrochemical
rGO	Reduced Graphene Oxide
SEM	Scanning Electron Microscopy
SiNW	Silicon Nanowire
TCN	Tubular Carbon Nitride
TEOA	Triethanolamine
TNTA	TiO_2_ Nanotube Arrays
TVD	Thermal Vapor Deposition
VB	Valance Band
XRD	X-ray Diffraction
